# *Plasmodium berghei* Cap93, a novel oocyst capsule-associated protein, plays a role in sporozoite development

**DOI:** 10.1186/s13071-017-2337-8

**Published:** 2017-08-25

**Authors:** Hanae Sasaki, Harumi Sekiguchi, Makoto Sugiyama, Hiromi Ikadai

**Affiliations:** 1 Hokusan Co. Ltd., 27-4, Kitanosato, Kitahiroshima, Hokkaido 061-111 Japan; 2 Laboratory of Veterinary Parasitology, School of Veterinary Medicine, Kitasato University, Towada, Aomori, 034-8628 Japan; 3 Laboratory of Veterinary Anatomy, School of Veterinary Medicine, Kitasato University, Towada, Aomori, 034-8628 Japan

**Keywords:** *Plasmodium berghei*, oocyst, PbCap93, Sporozoite, Knocked out parasite

## Abstract

**Background:**

Disruption of *Plasmodium* oocyst capsule protein (PbCap380), and an oocyst wall interior protein, circumsporozoite protein, results in sporozoites not being formed, despite the formation of oocysts, and prevents malaria transmission. Therefore, these key oocyst capsule-associated proteins are responsible for the development of the oocyst capsule and play an important role in the later growth and maintenance of sporozoites. We attempted to discover novel oocyst capsule-associated proteins and analyze their functions by assuming that such proteins will be strategically important targets for preventing malaria transmission. A putative, novel oocyst capsule-associated protein, known as PbCap93, was determined from the PlasmoDB database, and we aimed to create a knockout parasite of the PbCap93 gene to analyze its functions in the mosquito stage.

**Results:**

To investigate the kinetics of PbCap93 protein expression, we labelled the asexual stage and mosquito stage parasites with anti-PbCap93 antibodies using IFAT. PbCap93 was detected in oocysts on day 15 after infection, though it was not detected in sporozoites of ruptured oocysts. PbCap93 localizes interior to the oocyst capsule alone without localization to the sporozoite plasma membrane. To gain further insight regarding PbCap93 function, we disrupted the gene in *P. berghei* parasites. Between 14 and 15 days after receiving a parasite-laden blood meal, 100 midguts were dissected from mosquitoes that received either wild-type (WT) or knocked out (KO) parasites. For WT parasites, the oocyst infection rate was 50%, whereas, for KO parasites, the infection rate was 16.7%. The average number of oocysts per midgut was 12 for the WT parasites compared with 0.8 for the KO parasites. Furthermore, KO parasite oocysts were significantly smaller than WT parasite oocytes. Using transmission electron microscopy, we observed that the electron density of the PbCap93-KO oocyst capsule was lower than that of the WT oocyte capsule.

**Conclusions:**

We posited that the PbCap93 protein is secreted from sporoblasts within the oocysts until sporozoites are formed. PbCap93 constructs the interior of the oocyst capsule or part of the plasma membrane and affects sporozoite differentiation. Further studies are warranted to understand the mechanism of oocyst formation.

**Electronic supplementary material:**

The online version of this article (doi:10.1186/s13071-017-2337-8) contains supplementary material, which is available to authorized users.

## Background

Along with acquired immune deficiency syndrome and tuberculosis, human malaria is one of the major infectious diseases in the world [1, 2]. The disease affects 270 million people, with 627,000 deaths per year. Mortality primarily affects children aged ≤5 years [3, 4]. Malaria is caused by the protozoan parasites, *Plasmodium* spp. in human blood and are transmitted human-to-human via insect vectors, the *Anopheles* mosquitoes. When an *Anopheles* mosquito bites a host, the parasites in their sporozoite form enter the blood stream and ultimately migrate to the host liver. In the liver, sporozoites develop into merozoites that are released into the blood circulation and then start a cycle of asexual reproduction. In blood, only a small portion of the parasites exist as gametocytes. When a host is bitten by a female *Anopheles* mosquito, gametocytes present in the host blood are ingested into the mosquito midgut, where the gametocytes differentiate into male and female gametes, which in turn form zygotes after fertilization. They then differentiate into motile ookinetes. The ookinetes migrate and traverse the midgut epithelium and lodge under the basement membrane to differentiate into oocysts at approximately 24–30 h after blood ingestion. Several thousands of sporozoites form within each oocyst, from which they are released approximately 2 weeks after blood ingestion [5–9]. The released sporozoites then migrate and invade the salivary glands of the mosquito, thereby enabling the transmission when this mosquito bites another host.

In *Plasmodium*, parasite differentiation in the mosquito is complex and undergoes severe losses at the oocyst stage. Of hundreds of ookinetes that form in the midgut, only a few differentiate into oocysts [10–15]. In the developing oocyst, nuclear division occurs in the absence of cell division resulting in a syncytium containing thousands of nuclei embedded in a common cytoplasm. The oocyst is surrounded by an inner plasma membrane and a rigid outer capsule [16–18]. The oocyst capsule makes sporozoite formation and its growth inside the capsule possible [18–21]. It is conjectured that the formation of the oocyst capsule stabilizes the growth of oocysts, thereby enabling their long-lasting presence within the mosquito’s hemocoel. Previous studies have revealed that deletion of an oocyst capsule surface *P. berghei* protein gene (PbCap380), and of an oocyst capsule interior protein gene, circumsporozoite protein (CSP), results in interruption of sporozoite differentiation [16, 22–25]. Therefore, these key oocyst capsule-associated proteins are not only responsible for the development of the oocyst capsule but also play an important role in their later growth and maintenance of sporozoites. In other words, they are essential for the survival, proliferation, and transmission of *Plasmodium* parasites.

In the present study, we identify and characterize a novel oocyst capsule-associated protein 93 of *Plasmodium berghei* (PbCap93), PbANKA_0905200.

## Methods

### Bioinformatics

The PbCap93 (PbANKA_0905200) genomic sequences used in this study were retrieved from PlasmoDB (http://​www.​plasmodb.​org). All *Plasmodium* orthologues are encoded by a single exon (Additional file 1).

### Mice, parasites, and mosquitoes

For *P. berghei* infections, 6- to 8-week old male BALB/c mice (SLC, Japan) were infected with wild-type *P. berghei* (ANKA strain 2.34). *Anopheles stephensi* (STE2 strain) mosquitoes were maintained at 27 °C and 80% relative humidity with a 14/10 h light/dark cycle in an insectary and fed 10% (*w*/*v*) sucrose solution.

### PbCap93 gene expression analysis

To study PbCap93 expression during the *P. berghei* life-cycle, using the Trizol reagent (Thermo Fisher Scientific, MA, USA), total RNA was isolated from blood samples obtained during the asexual stage of *P. berghei*-infected mouse at 10% parasitemia and from those obtained during the mosquito stages at 30 min, 1 day, 3 days, 5 days, 10 days, 15 days and 17 days after infection. cDNA was synthesized using the ReverTra Ace kit (Toyobo, Osaka, Japan). PCR was performed using gene-specific primers PbCap93 (PbCap93-F: 5′-CCT GCT TCT TCA ACA GAT TAT AAT G-3′ and PbCap93-R: 5′-GGA TTG TTT GAA ATC GAA TCG AAA G-3′), PbCap380 (PbCap380-F: 5′-GAA ATC ACC ATT TAA TTT CTC CAA TGG GT-3′ and PbCap380-R: 5′-TGT AGT TCG AAA AGG ATG GTT TTG ATT GT-3′ [26]), CHT1 (CHT1-F: 5′-GAT TGG GAA CCA AAT GGA AGT TTT AAC-3′ and CHT1-R: 5′-GAT ACA CAT GAT AAT GCT GCA TTT GAT G-3′), and CSP (CSP-F: 5′-GAC CCA GCA CCA CCA AAC GCA AAT G-3′ and CSP-R: 5′-CCT TGT GGT GGT GCT GGG TCA TTT G-3′ [27]). *Plasmodium* 18S ribosomal RNA (18S rRNA) (18S rRNA-F: 5′-AAG CAT TAA ATA AAG CGA ATA CAT CCT TAC-3′ and 18S rRNA-R: 5′-GGA GAT TGG TTT TGA CGT TTA TGT G-3′) was used for normalization [28].

### Immunization and anti-PbCap93 serum

Polyclonal antibodies were raised against the PbCap93 protein (amino acids 379–392) (Additional file 1) and PbCSP (PBANKA_040320; amino acids 133–148) and used in this study (Eurofins Genomics Inc., Tokyo, Japan).

### Indirect immunofluorescence assay

To detect PbCap93 in oocysts, infected midguts were fixed in acetone/methanol for 1 h and then blocked in phosphate-buffered saline (PBS) containing 1% bovine serum albumin (BSA). Midguts were incubated with rabbit anti-PbCap93 sera (1:200) at 37 °C for 60 min, washed three times with PBS, and then incubated with Alexa fluor 488 goat-conjugated anti-rabbit immunoglobulin G (IgG) (1:500, Thermo Fisher Scientific) at 37 °C for 30 min. Pre-immune serum from the same rabbit was used as a control. Oocysts were observed after staining with anti-PbCap380 [26] (1:400) and anti-PbCSP (1:400) antibodies and detected with Alexa fluor 568 goat-conjugated anti-rabbit secondary antibody (Thermo Fisher Scientific). *Plasmodium berghei* sporozoites in oocysts and salivary glands were fixed in acetone/methanol and processed as described above.

### Confocal microscopy

Midguts were dissected 10 days after infection and processed as described above. Paraffin-embedded *P. berghei*-infected midgut tissue sections were deparaffinized using xylene and graded alcohols. The slides were incubated in 10% BSA in PBS for 1 h and then incubated overnight with primary antibodies at room temperature. Rabbit antibodies against PbCap93 diluted in 0.1% BSA in PBS (1:400) were used as primary antibodies. Preimmune rabbit sera (diluted 1:100) were used as negative controls. After washing three times with PBS, the slides were incubated with Alexa fluor 488 goat-conjugated anti-rabbit IgG (1:800 dilution) (Thermo Fisher Scientific). Antibodies against PbCap380 and PbCSP were used for oocyst double-labelling experiments and were detected using Alexa fluor 568 goat-conjugated anti-rabbit secondary antibodies. *P. berghei* nuclei were counterstained with SlowFade Diamond Antifade Mountant with DAPI (4′, 6-diamidino-2-phenylindole) (Thermo Fisher Scientific). Sections were observed by confocal microscopy (LSM 710, Carl Zeiss Inc., Oberkochen, Germany).

### Generation of PbCap93-KO parasites

PbCap93 was knocked out (KO) using double-crossover homologous recombination technology as previously described [29]*.* For targeted disruption of the PbCap93 locus, a disruption plasmid was generated by amplification of a PCR fragment using primers P93F (5′-GAT GTT ATG TGG TAT GTT CCA GA-3′) and P93R (5′-CAT TTT GGA AAT GTG TAA TGC TCA-3′) and *P. berghei* genomic DNA as a template. The gene was cloned into the pCR-BluntII-TOPO vector (Thermo Fisher Scientific), resulting in the plasmid pCap93. Subsequently, pCap93 was digested using *Acc* I. Digested pCap93 was inserted into the *hdhfr* expression cassette [30]. pCap93 (10 μg) was linearized with *Pvu*II and electroporated into cultured *P. berghei* schizonts using Nucleofector II. Transfected parasites were intravenously injected into male BALB/c mice and treated with pyrimethamine (70 μg/ml) 24 h later via drinking water. Infected blood was collected to examine the integration of the disruption cassette by PCR using integration-specific primers P93R and DHFR-F1 (5′-CTT CTC TGT GTA TTA ATA TTG T-3′) and P93F and DHFR-R1 (5′-CTA TCA ATT ATT TCC CGT GG-3′). Parasites were cloned by limiting dilution.

### Phenotypic analysis of PbCap93-KO parasites

Development of wild-type (WT) and PbCap93-KO parasites in mice was assessed by injecting a known number of parasites in BALB/c mice. Eight mice each were injected with 1 × 10^6^ PbCap93-KO parasites, whereas the other seven were injected with 1 × 10^6^ WT parasites. Parasitemia was monitored daily via Giemsa-stained blood smears. For 10% parasitemia, gametocytaemia (gametocytes per 500 red blood corpuscles) and gametocyte sex ratio (in 300 mature gametocytes) were determined by Giemsa-stained tail blood smears. Exflagellation of male gametocytes was quantified as previously described [31, 32]. Briefly, 2 μl of gametocyte-infected blood was obtained from the tail vein and mixed immediately with 38 μl of complete ookinete culture medium. The mixture was placed under a coverslip at RT, and 5 min later, exflagellation centres were counted over the next 10 min using a phase contrast microscope. The ability of parasites to differentiate into gametocytes and from male gametes (exflagellation) was assessed. Infected mice were fed to *A. stephensi* mosquitoes, and oocysts (days 14–15) were microscopically examined. For each mouse, up to 30 mosquitoes were dissected 14–15 days after feeding. The midguts were stained with 0.5% mercurochrome (Merck Millipore, MA, USA). Oocysts were counted to determine the prevalence of infection (number of infected mosquitoes) and intensity of infection (number of oocysts per positive midgut) [33].

### Transmission electron microscopy

Mosquito midguts at 15 days post-infection were fixed in 2.5% glutaraldehyde and 2% paraformaldehyde in 0.05 M sodium cacodylate buffer (pH 7.4) at 4 °C for 2 h. After rinses, samples were post-fixed at RT in buffered 1% osmium tetroxide for 2 h and then dehydrated in ethanol and propylene oxide series and embedded in epoxy resin. Ultrathin sections were cut using an Ultracut N (Reichert-Nissei, Tokyo, Japan), stained using uranyl acetate followed by lead citrate. Sections were examined using a H-7650 transmission electron microscope (Hitachi Ltd., Tokyo, Japan).

### Statistical analyses

A Student’s *t*-test was used to statistically compare the groups (parasitemia, gametocytaemia, and oocyst size). The intensity of infection (number of oocysts/midguts) was analyzed using the Mann–Whitney U-test. For these statistical analyses, GraphPad Prism software (GraphPad Software, Inc., CA, USA) was used. *P* < 0.05 was considered statistically significant.

## Results

### Identification of the gene encoding *P. berghei* capsule-associated protein 93 (PbCap93)

To select candidates for novel oocyst capsule-associated proteins, we used a *P. bergehi* in PlasmoDB database (http://plasmodb.org/plasmo/). The following criteria were used: “expressed only in the oocyst stage,” “possessing transmembrane domains,” and “conserved proteins with unknown function”. Using these criteria, we identify PbCap93 (PBANKA_0905200), which is highly homologous with other protozoa. A total of 65 genes were found only to be expressed in the oocyst stage, of which 14 genes have transmembrane domains and of these, five genes that encoded novel conserved proteins with unknown functions. Finally, among these five genes, those having high homology with other protozoa were selected.

**Fig. 1:**
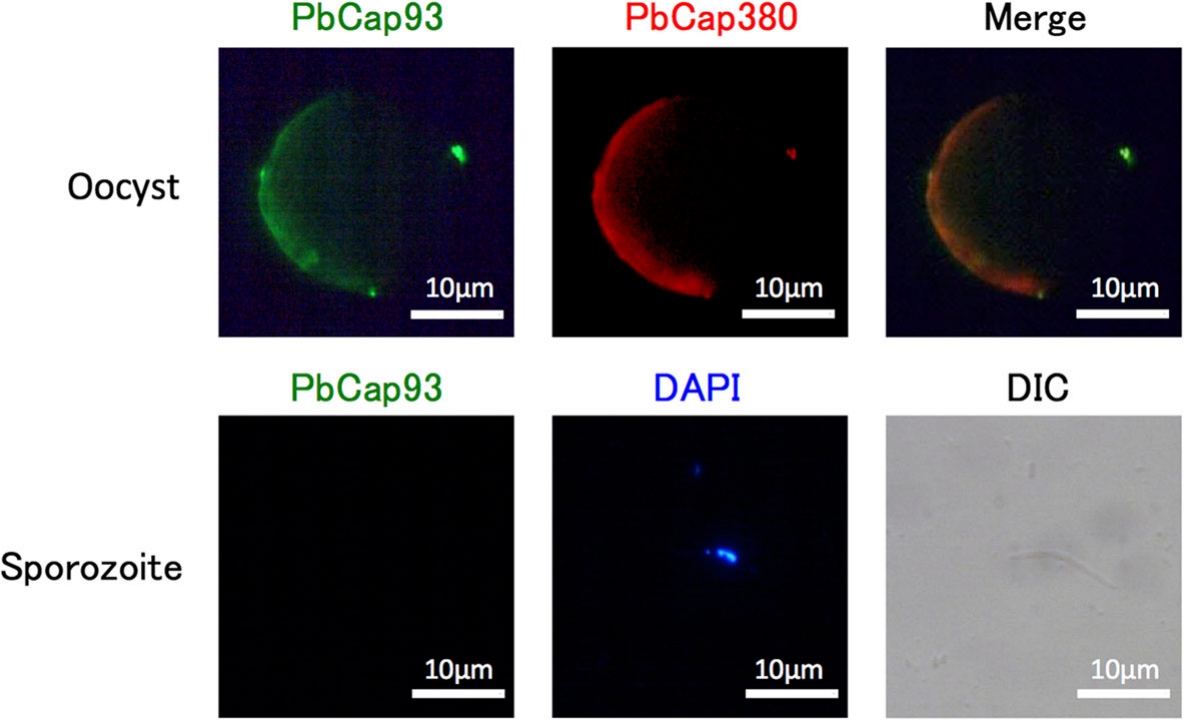
Subcellular localization of PbCap93. Immunofluorescence assays of oocysts at 15 days post-infection (upper panels) and sporozoite (lower panels) with PbCap93 (green) and PbCap380 (red) antibodies, stained with the DAPI nuclear stain (blue), and showing differential interference contrast (DIC), as indicated. *Scale-bars*: 10 μm

PbCap93 has orthologues in every sequenced *Plasmodium* species. The *P. berghei*-predicted amino acid sequence was determined to be > 69% identical to the *P. chabaudi-* (89.3%), *P. yoelii-* (92.5%), *P. falciparum-* (69.9%), *P. knowlesi-* (69.0%), and *P. vivax-*predicted sequences (77.0%) (Fig. 1; PlasmoDB accession numbers: PCHAS_0706400, PY17X_0906600, PF3D7_1143800, PKNH_0941700, and PVX_092795, respectively). The C-terminal half of the orthologues is more similar than the N-terminal half (Additional file 1).

### PbCap93 is expressed during oocyst development

**Fig. 2:**
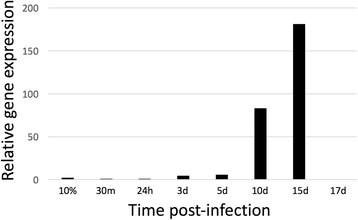
Quantification of PbCap93 mRNA abundance. Quantitative RT-PCR was performed using cDNA prepared from blood stage parasites at 10% parasitemia (10%) and midguts dissected at different times after infection (30 min to 17 days, d). Experiments were performed in triplicate, and data shown are from one representative experiment. Four independent biological replicates were performed for each experimental condition

To investigate the kinetics of PbCap93 protein expression, we labelled asexual stage and mosquito stage parasites with anti-PbCap93 antibodies by IFAT. PbCap93 was not detected in blood stage but was detected in oocysts on day 15 after infection (Fig. 1). Quantitative RT-PCR (qRT-PCR) analysis indicated that PbCap93 is expressed during oocyst development, particularly in late oocysts but its expression diminished by day 17 when the sporozoites within the oocysts matured (Fig. 2). The antibody did not detect the protein in oocysts sporozoites or salivary gland sporozoites. (Fig. 1).

### PbCap93 localizes internally to the oocyst capsule and may be associated with the oocyst capsule

**Fig. 3:**
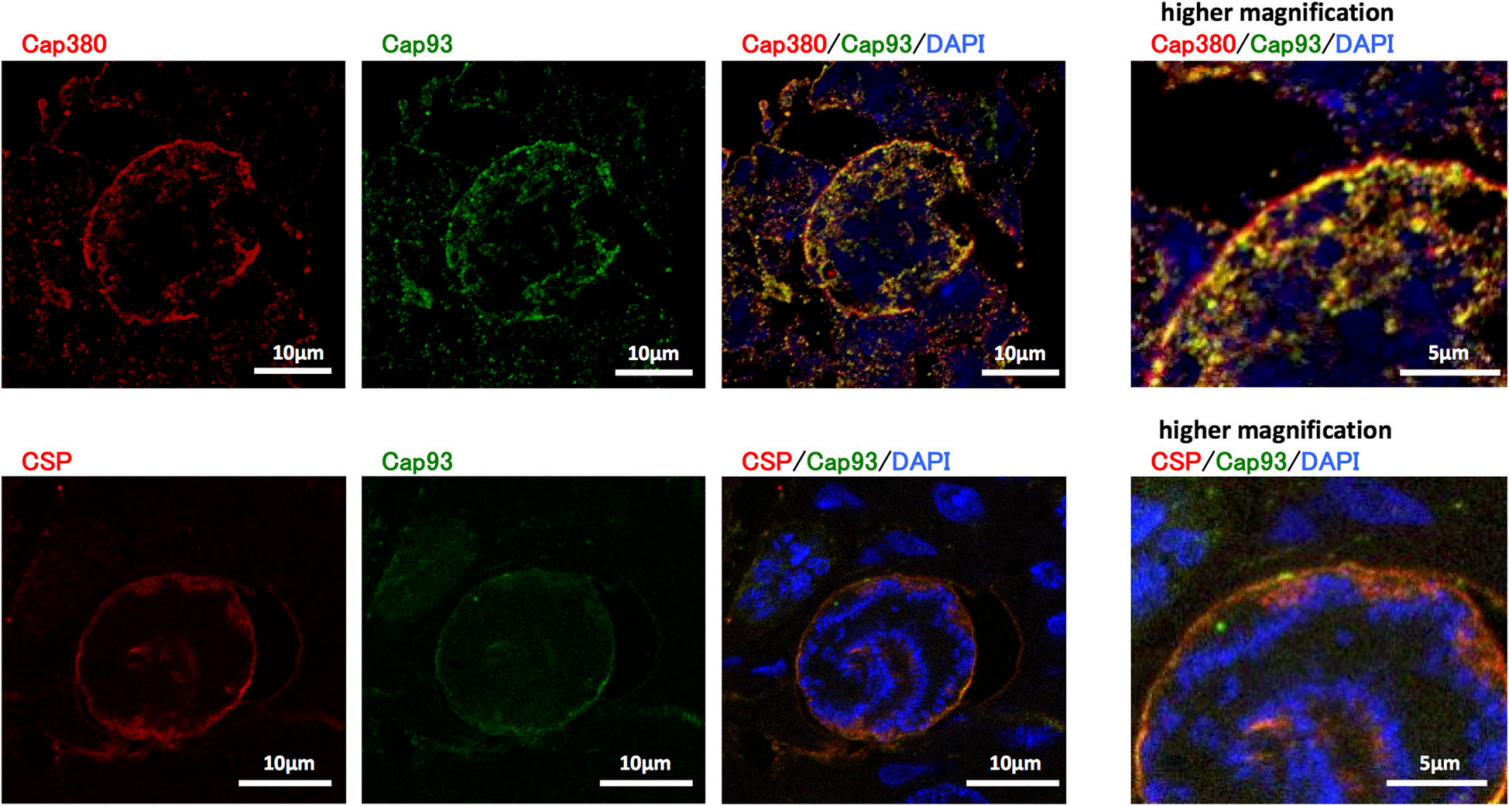
PbCap93 localizes to the oocyst capsule interior. Confocal microscopy of a 15 days post-infection oocyst. The parasite was double labelled with antibodies against PbCap380 (red: upper panel), PbCSP (red: lower panel), and PbCap93 (green). The right panels show a higher magnification of the area in the merged image. PbCap93 appears to be located internally relative to PbCap380 and co-localized to CSP

PbCap380 is a surface protein of the oocyst capsule [24]. PbCSP, the most abundant sporozoite plasma membrane protein is also localized on the inner face of the oocyst capsule [23, 25, 34]. Confocal microscopy of day 10 oocysts using anti-PbCap93, anti-PbCap380, and anti-PbCSP antibodies indicated that PbCap93 co-localizes with PbCSP and PbCap380 (Fig. 3). Moreover, PbCap93 was not detected in sporozoites of oocysts and salivary glands (Fig. 1). Therefore, our data suggested that PbCap93 localizes to the oocyst capsule alone without localizing to the sporozoite plasma membrane.

### Production of PbCap93-KO parasites

**Fig. 4:**
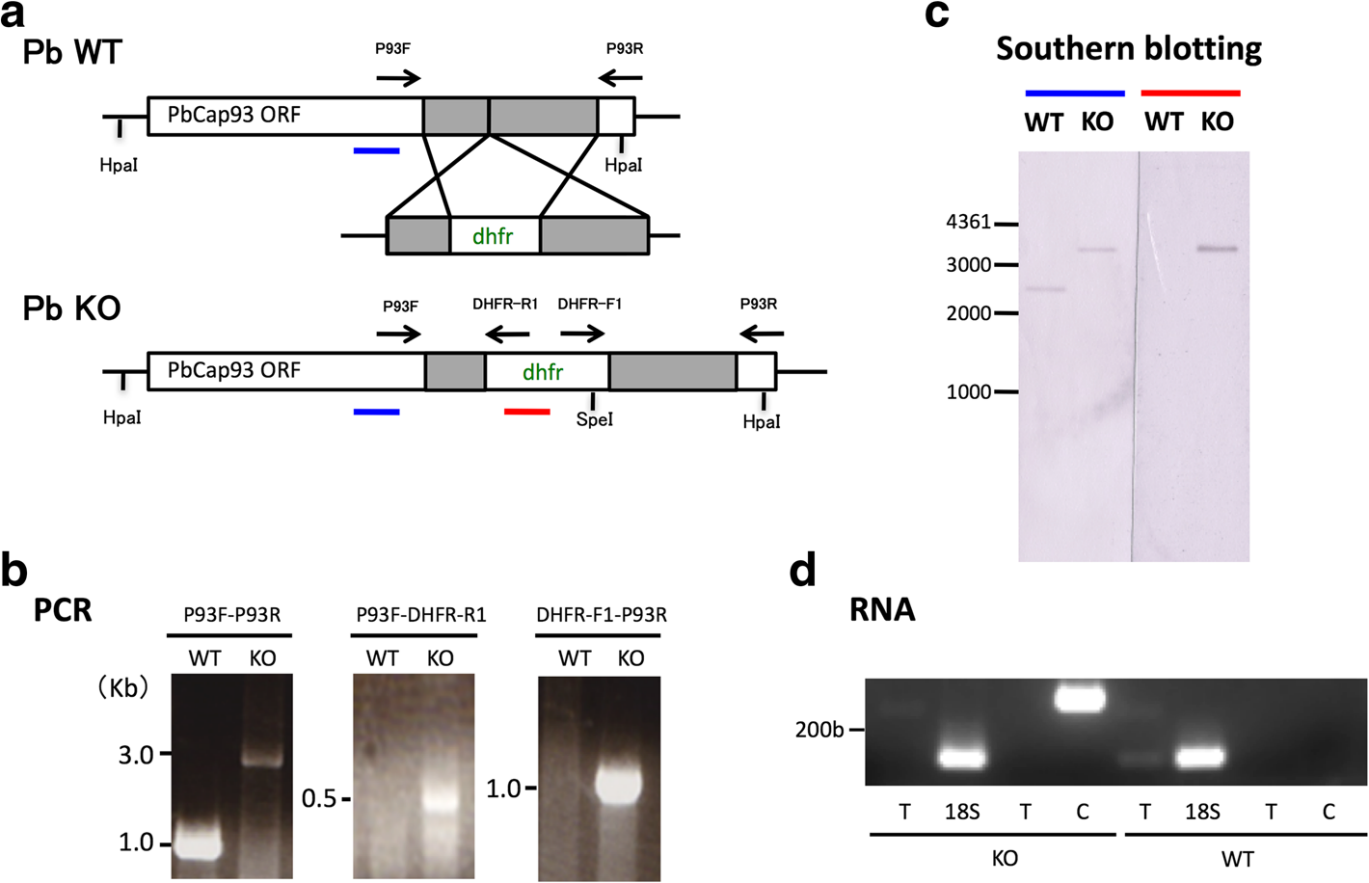
Characterization of PbCap93 KO parasites. **a** Schematic of targeted disruption of the PbCap93 gene. The targeting vector (middle) containing a selectable marker gene was integrated into the PbCap93 gene locus (top) by double crossover. This recombination event resulted in the disruption of the PbCap93 gene and conferred pyrimethamine resistance (bottom). **b** PCR analysis of wild-type (WT) and PbCap93-KO parasites. Integration-specific PCR primer sets were used: P93F and P93R, P93F and DHFR-R1, and P93R and DHFR-F1. **c** Genomic Southern blot hybridization of WT and PbCap93-KO parasites. Parasite genomic DNA was digested with *Spe*I/*Hpa*I and hybridized with the probe indicated by a solid bar (blue and red) in **a**. By integration of the targeting construct, the size of detected fragments increased from 2.5 to 3.5 kbp (blue) and 3.5 kbp (red). **d** Semi-quantification of PbCap93 mRNA abundance. Semi-quantitative RT-PCR was performed using cDNA prepared from midguts of mosquitoes infected with either WT or PbCap93-KO parasites at 10 days post-infection

To gain further insight into PbCap93 function, we disrupted the gene in *P. berghei* parasites (Fig. 4a). Independent clonal parasite lines generated from the transfection were confirmed for gene disruption by insertion-specific PCR. To ascertain that the drug-resistance gene hDHFR was successfully inserted into the KO parasites, we performed PCR using the following three primer combinations: P93F-P93R, P93F-DHFR-R1, and DHFR-F1-P93R. As a result, we confirmed amplification products with their expected size from the P93F-P93R, P93F-DHFR-R1, and DHFR-F1-P93R primer combinations, respectively. In WT parasites, the P93F-P93R primer combination yielded an amplification product measuring 1066 bp, whereas the other two primer combinations did not produce any amplification products (Fig. 4a, b). Moreover, Southern blot analysis confirmed that the drug-resistance gene was successfully inserted into the targeted P93F–P93R region and semi-quantitative RT-PCR ascertained that no mRNA was being expressed. Consequently, the PbCap93-KO was successfully generated (Fig. 4c, d).

### PbCap93 is not required for asexual growth but is crucial for oocyst development

**Fig. 5:**
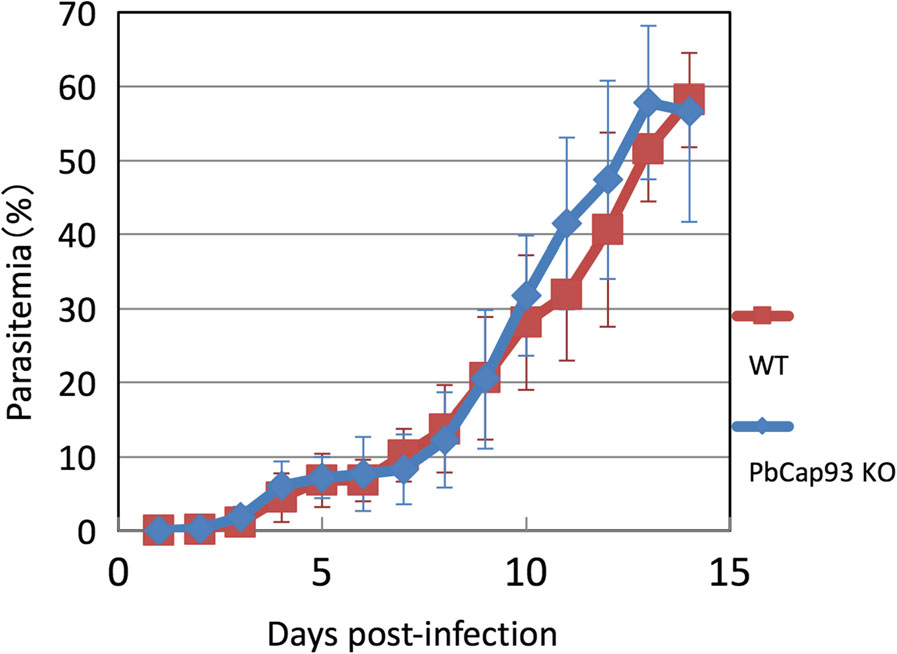
Comparison of parasitemia in mice infected with either WT or PbCap93-KO parasites. Comparison of parasitemia in mice intravenously infected with WT (red) or PbCap93-KO (blue) parasites. All mice were infected by intravenously injecting 10^6^ blood-stage parasites. Number of mice analyzed: *n* = 8 (WT) and *n* = 7 (KO)

**Fig. 6:**
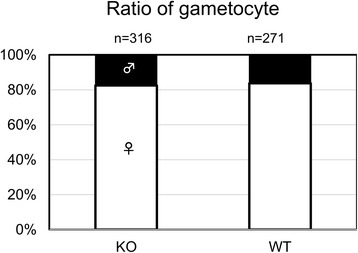
The proportion of female and male gametocyte in mice infected with either WT or PbCap93-KO parasites. The proportion of female (white) and male (black) gametocytes in mice intravenously infected with WT and PbCap93-KO parasites. Number of gametocytes analyzed: *n* = 316 (WT) and *n* = 271 (KO)

PbCap93 is expressed in the mosquito stages of parasite development, and it is expected not to be required for asexual growth in mice. When mice were infected with either PbCap93-KO parasites or WT parasites, no significant changes were observed by day 14 (Fig. 5). Furthermore, when blood smears of KO parasite-infected and WT parasite-infected mice were compared by Giemsa staining, no morphological differences were apparent between KO and WT parasites (data not shown). Neither gametocytaemia nor female/male gametocyte ratio was affected (Table 1, Fig. 6).

**Table 1:** Ratio of gametocytemia in 10% parasitemia

Parasite	♀-gametocytes/infected RBC	♂-gametocytes/infected RBC
	Mean ± SD	Mean ± SD
WT	7.4 ± 3.9	1.4 ± 1.0
KO	7.1 ± 2.4	1.5 ± 0.7

*Abbreviations*: *KO* knocked out, *RBC* red blood cell, *WT* wild-type, *SD* standard deviation

**Fig. 7:**
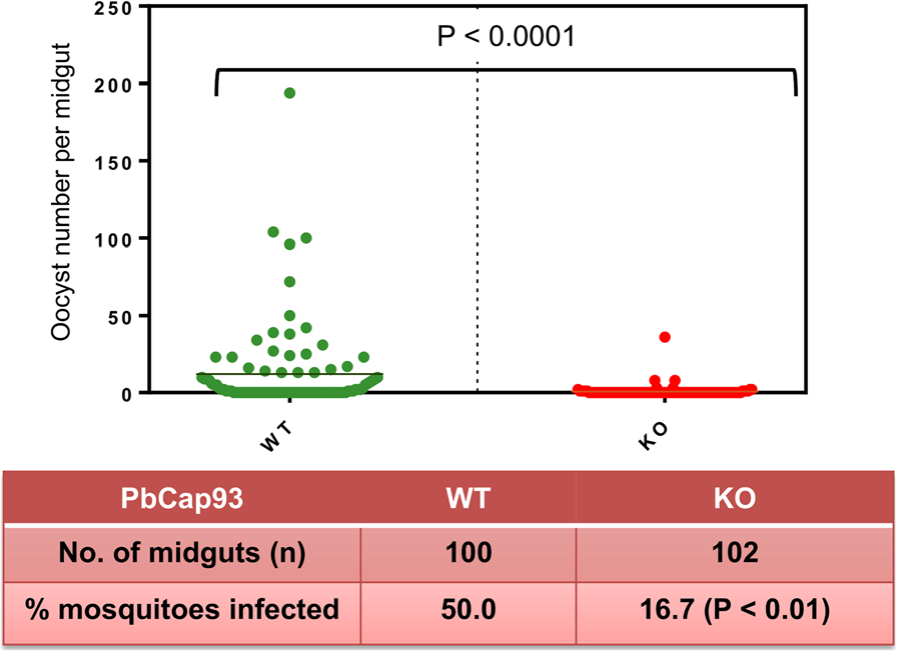
PbCap93 is important for oocyst development. Oocyst number per midgut of mosquitoes infected with either WT or PbCap93-KO parasites. Horizontal bars represent medians. Data from three independent replicates. % mosquitoes infected: proportion of mosquitoes carrying at least one oocyst

**Fig. 8:**
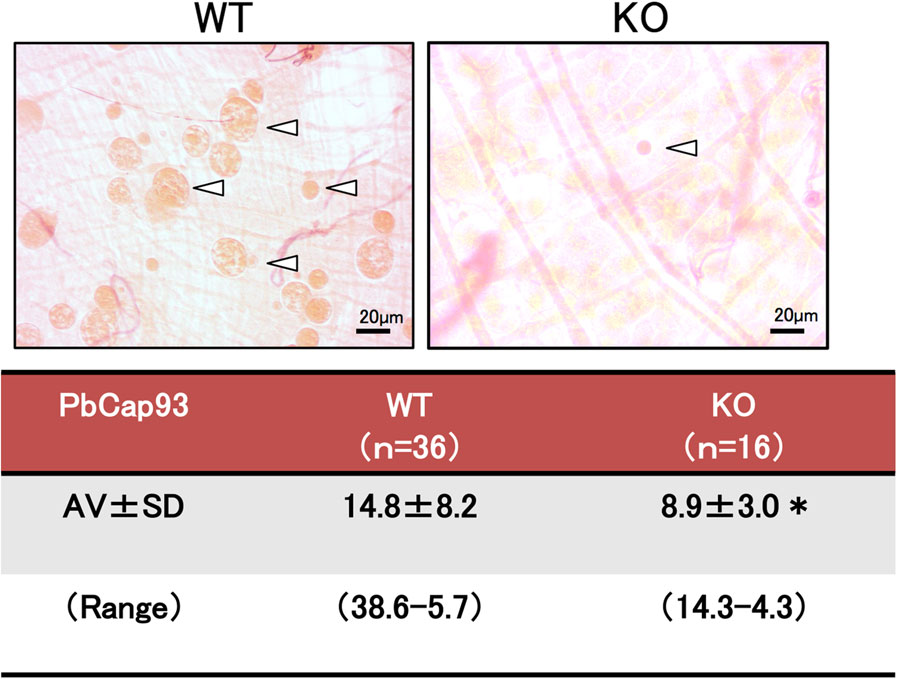
Representative oocyst size and numbers in WT and PbCap93-KO parasites. Diameters (μm) of 14 days post-infection WT and PbCap93-KO oocysts (arrowheads). *P < 0.01. *Scale-bar*: 20 μm

Between 14 and 15 days after receiving a parasite-laden blood meal, 100 midguts and 102 midguts were dissected from *A. stephensi* mosquitoes that received WT or KO parasites, respectively. For WT parasites, the oocyst infection rate was 50%, with 50 midguts harbouring oocysts. For KO parasites, on the other hand, the infection rate was 16.7%, with only 17 midguts harbouring oocysts (Fig. 7). The average number of oocysts per midgut was 12 for the WT parasites compared with 0.8 for the KO parasites. Meanwhile, KO parasite oocysts were significantly smaller than WT parasite oocytes (Fig. 8).

**Fig. 9:**
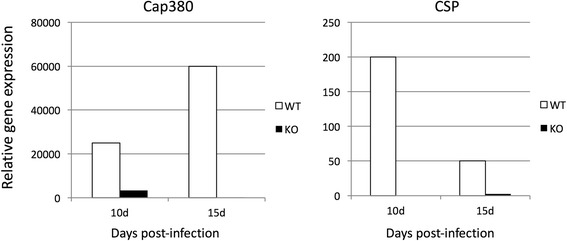
Quantification of PbCap380 and CSP mRNA in PbCap93-KO parasites. qRT-PCR was performed using cDNA prepared from midguts dissected after infection (10 and 15 days post-infection). Experiments were performed in triplicate, and data shown are from one representative experiment. Four independent biological replicates were performed for each experimental condition

Subsequently, we examined by real-time PCR the expression dynamics of PbCap380 and CSP in mosquitoes infected with WT parasites. As a result, mRNA abundance of both genes peaked between 10 and 15 days post-infection. Similarly, we also observed dynamics of PbCap380 and CSP genes in mosquitoes infected with PbCap93-KO parasites, and we found that PbCap380 and CSP were substantially suppressed at 10 and 15 days post-infection (Fig. 9).

**Fig. 10:**
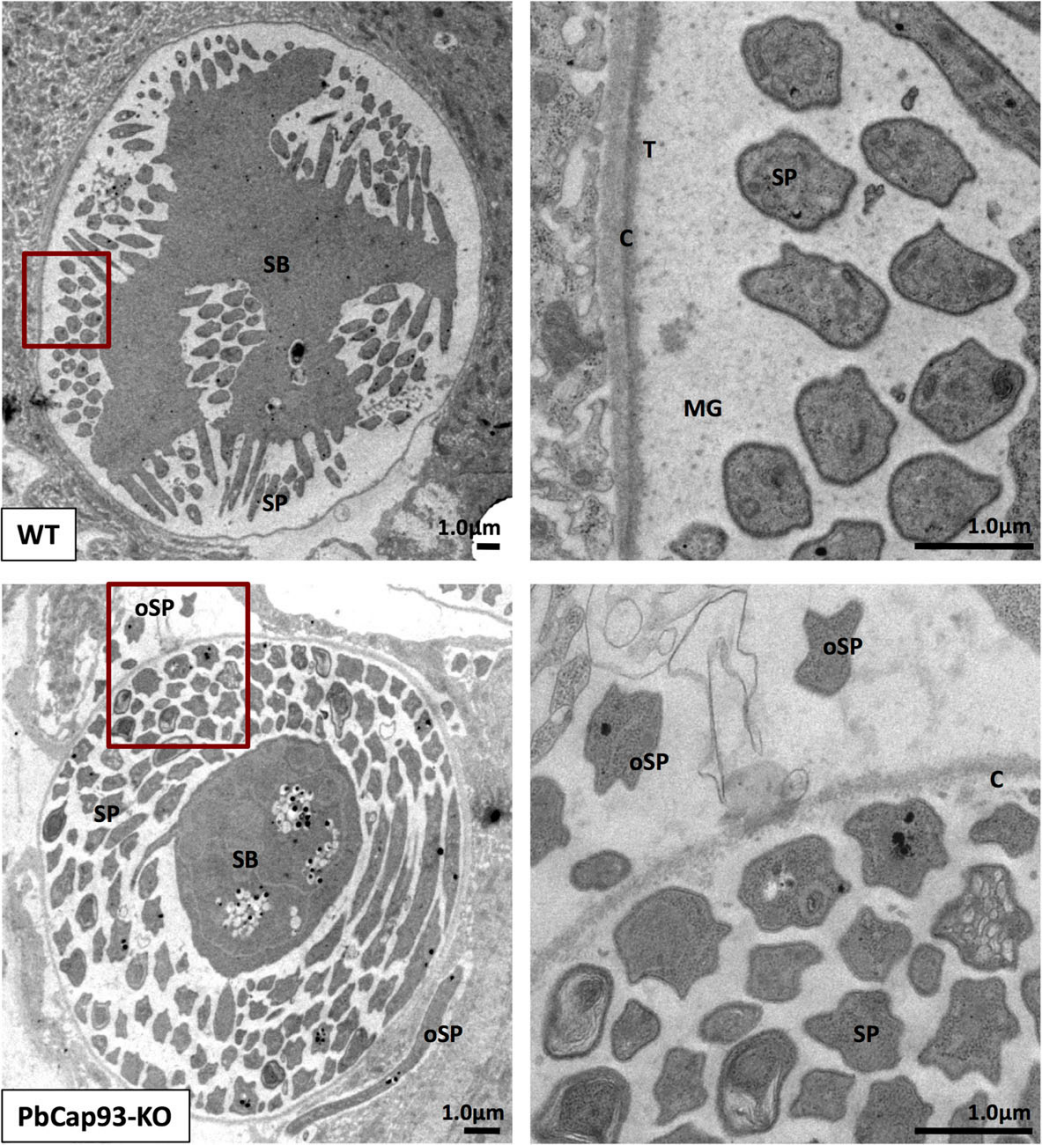
Ultrastructure of oocyst development in PbCap93-KO parasites. Left panels: Low power ultrastructural images of oocyst development at 15 days post-infection. Right panels: Details of the oocyst capsule from the area outlined by red squares on the left. The cleft enlarges, dividing the oocyst cytoplasm into the intermediate sporoblast form (SB) and budding sporozoite (SP). Some sporozoites are outside the oocyst (oSP). Small tufts (T) of material appear on the inner capsule (C) and matrix granules (MG). *Scale-bars*: 1 μm

By transmission electron microscopy, we observed that the electron density of the PbCap93-KO oocyst capsule is lower than that of WT parasites. Accordingly, KO oocysts had fewer matrix granules (Fig. 10, MG), which were abundant in the WT oocyst capsule. Moreover, the cross-section structures of sporozoites in KO oocysts varied significantly in size and cell structure, with some taking an amorphous shape, compared with sporozoites in WT oocysts (Fig. 10). Some sporozoites of KO parasites were observed outside the oocyst (Fig. 10, oSP).

## Discussion

The oocyst capsule is in contact with the basal lamina of the mosquito midgut epithelium. The oocyst capsule helps to fend off defensive responses by the mosquito. It is intriguing how this supposedly rigid structure enlarges during the dramatic growth of the oocyst. This may happen by intercalation of newly synthesized proteins or by protein addition at one specific growth site. The deficiency of a known oocyst wall surface protein, PbCap380, and an oocyst capsule interior protein, CSP, results in sporozoites not being formed despite the formation of oocysts [16, 22–25]. Therefore, these key oocyst capsule-associated proteins are not only responsible for the development of the oocyst capsule but also play an important role in their later growth and maintenance of sporozoites. In the present study, we sought to search for novel oocyst capsule-associated proteins, and analyze their functions on the assuming that such proteins will prove to be important targets for preventing the transmission of malaria.

PbCap93 is expressed in oocysts but not by blood stage parasites, and PbCSP appears to be integrated into the oocyst capsule alone without association with sporozoite plasma membrane. We gleaned that PbCap93 is probably secreted from sporoblasts within the lining of the oocyst capsule. Therefore, the fewer number of protein granules observed within the oocysts was attributed to knocking out of PbCap93. This phenomenon was considered to be due to the abnormal differentiation of sporoblasts, which made it difficult for sporozoites to secrete PbCap93. Accordingly, the lack of PbCap93 constituting the oocyst capsule led to the low electron density of the oocyst capsule. PbCap380 is a protein expressed in the exterior oocyst capsule. It is abundantly expressed at the later stage of oocyst formation [24]. However, based on the assessment of mRNA abundance, PbCap380 expression is regulated in PbCap93-KO parasites. This may have resulted in the insufficient composition of the oocyst capsule, thereby resulting in the observed lower electron density.

CSP is expressed during sporozoite division at the oocyst formation [23, 25]. Because CSP mRNA abundance was almost undetectable in PbCap93-KO oocysts, sporozoite division and maturation may have been adversely affected, with some shape deformities observed in sporozoites within the oocysts. Therefore, our data suggested that PbCap93 localizes to the oocyst capsule interior, into the inner face of the oocyst capsule, implying that the PbCap93 protein participates in the maturation of sporozoites in oocysts and/or at the time of capsule rupture.

## Conclusions

We posited that the PbCap93 protein is secreted from sporoblasts within the oocysts until sporozoites are formed. In addition, PbCap93 constructs the interior of the oocyst capsule or part of the plasma membrane and affects sporozoite differentiation. Further studies are needed to determine the potential role of the PbCap93 protein and the underlying mechanism of oocyst formation.

## Additional file

Additional file 1: Sequence comparison of predicted PbCap93 (PBANKA_0905200) orthologues. The *Plasmodium berghei*-predicted amino acid sequence available in the database was > 69% similar to the *P. falciparum*, 69.9%; *P. knowlesi*, 69.0%; *P. vivax*, 77.0%; *P. chabaudi*, 89.3%; and *P. yoelii*, 92.5% (PlasmoDB accession numbers: PCHAS_0706400, PY17X_0906600, PF3D7_1143800, PKNH_0941700, and PVX_092795, respectively). The similarity at the C-terminal half is higher than that in the N-terminal half. The red square indicates the predicted transmembrane (TM) sequence. The PbCap93 sequence used for generating antibody is denoted by the green square (Anti-PbCap93aa). This alignment was generated using the Genetyx software. Identical amino acid residues in all four *Plasmodium* species are indicated by black squares. *Abbreviations*: PCHAS, *P. chabaudi*; PY17X, *P. yoelii*; PF3D7, *P. falciparum*; PKNH, *P. knowlesi*; PVX, *P. vivax*. (TIFF 10501 kb)

## Abbreviations

CSP: Circumsporozoite protein
KO: Knocked out
PbCap93: oocyst capsule-associated protein 93 of *Plasmodium berghei*
WT: wild-type
