# Supplementary figures and images for: Plasmodium berghei Cap93, a novel oocyst capsule-associated protein, plays a role in sporozoite development
Source: Parasit Vectors. 2017 Aug 25;10:399. doi: 10.1186/s13071-017-2337-8 (PMC5574095; doi:10.1186/s13071-017-2337-8)

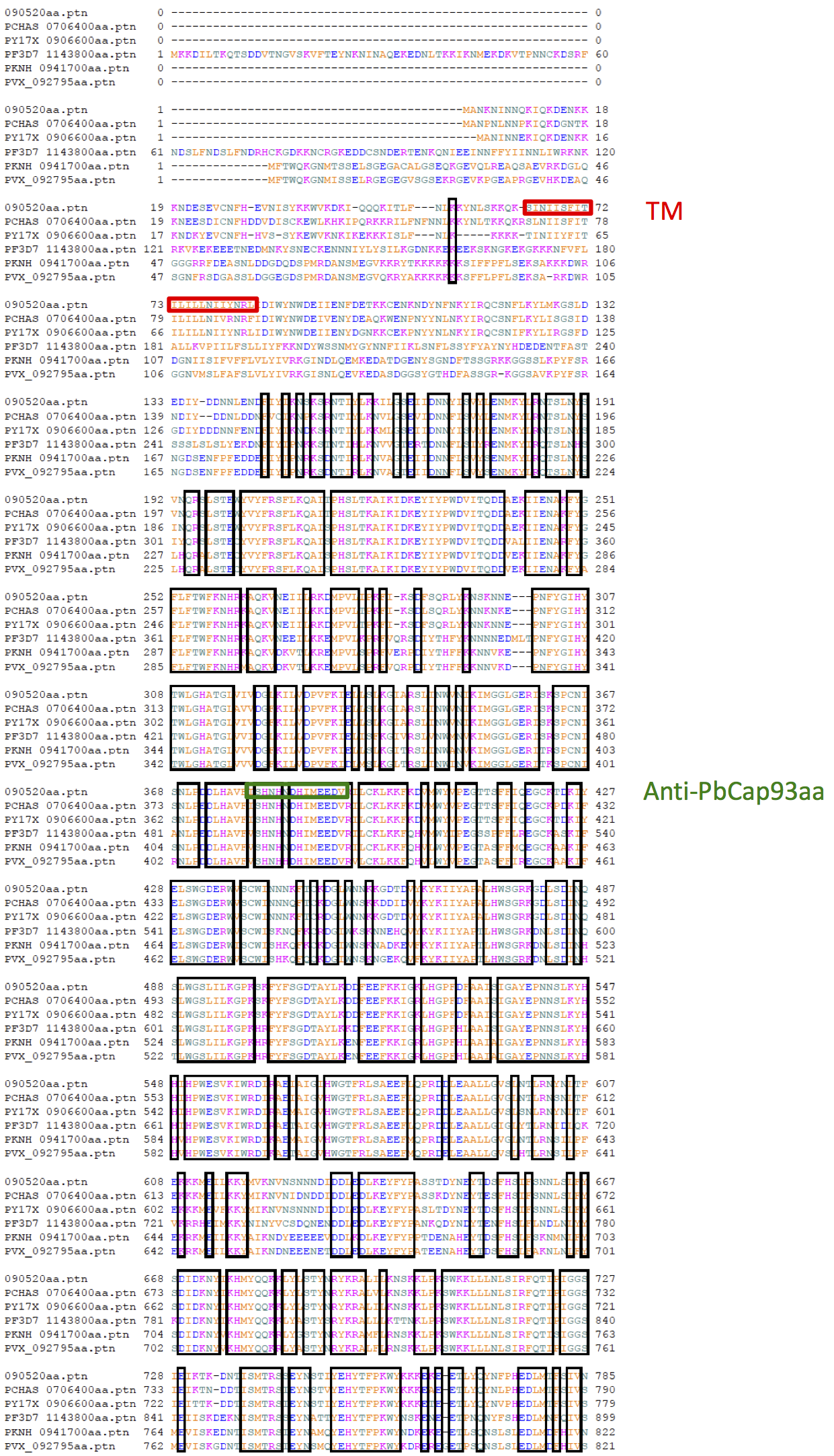

Supplement: Additional file 1: — Sequence comparison of predicted PbCap93 (PBANKA_0905200) orthologues. The Plasmodium berghei-predicted amino acid sequence available in the database was > 69% similar to the P. falciparum, 69.9%; P. knowlesi, 69.0%; P. vivax, 77.0%; P. chabaudi, 89.3%; and P. yoelii, 92.5% (PlasmoDB accession numbers: PCHAS_0706400, PY17X_0906600, PF3D7_1143800, PKNH_0941700, and PVX_092795, respectively). The similarity at the C-terminal half is higher than that in the N-terminal half. The red square indicates the predicted transmembrane (TM) sequence. The PbCap93 sequence used for generating antibody is denoted by the green square (Anti-PbCap93aa). This alignment was generated using the Genetyx software. Identical amino acid residues in all four Plasmodium species are indicated by black squares. Abbreviations: PCHAS, P. chabaudi; PY17X, P. yoelii; PF3D7, P. falciparum; PKNH, P. knowlesi; PVX, P. vivax. (TIFF 10501 kb) [file 13071_2017_2337_MOESM1_ESM.tif]
